# Lessons learned from the deployment of oral cholera vaccine by maintaining the controlled temperature chain (CTC) in an urban setting, Bangladesh

**DOI:** 10.1080/21645515.2026.2705004

**Published:** 2026-07-23

**Authors:** Md. Taufiqul Islam, Ashraful Islam Khan, Zahid Hasan Khan, Mohammad Ashraful Amin, Sabina Shahnaz, Quazi Fayeza Sultana, Md. Taufiqur Rahman Bhuiyan, Abhishek Rimal, Joanie Robertson, Anna-Lea Kahn, Firdausi Qadri

**Affiliations:** a International Centre for Diarrhoeal Disease Research Bangladesh (icddr,b), Dhaka, Bangladesh; b International Federation of Red Cross and Red Crescent Societies, Bangladesh Red Crescent Society, Dhaka, Bangladesh; c PATH, Seattle, USA; d Immunization, Vaccines and Biologicals Department, World Health Organization (WHO), Geneva, Switzerland; e Swiss Centre for International Health, Swiss Tropical Public Health Institute (Swiss TPH), Allschwil, Switzerland

**Keywords:** Bangladesh, cholera, controlled temperature chain, public health, vaccine

## Abstract

Cholera, caused by *Vibrio cholerae*, remains a significant public health issue, especially in regions prone to outbreaks. While the oral cholera vaccine (OCV) is an effective preventive tool, its use is often limited by the traditional cold chain requirements. The controlled temperature chain (CTC) provides an alternative by allowing vaccines to be stored at ambient temperatures for limited periods. This study explored participants’ hands-on experiences with CTC compared with standard cold chain (SCC). This qualitative study during the March 2024 OCV campaign in Dhaka, Bangladesh, used purposive sampling, including six in-depth interviews (IDIs) with volunteers and four key informant interviews (KIIs) with policymakers. Volunteers were selected from two groups (Group A: SCC, and Group B: CTC) based on their vaccination, supervision, and record-keeping roles, while the policymakers were involved in planning during campaign deployment. The interviews observed frontline experiences and expert opinions on the feasibility, appropriateness, and logistical difficulties of both approaches. Compared to the SCC, the CTC strategy provided operational advantages, including reduced transportation costs, decreased refrigeration dependency, and easier management in the hard-to-reach areas. Participants highlighted that the vaccines could be stored at ambient temperatures for up to 14 d under CTC, enabling extended deployment with minimal wastage. Overall, the participants perceived the CTC strategy as more competent in vaccine deployment, requiring fewer resources. For improving vaccine deployment strategies in cholera prevention, this study provided practical insights by using the CTC approach.

## Introduction

Cholera, an acute diarrheal disease caused by the bacterium *Vibrio cholerae*,^1^ poses a significant public health threat, especially in areas with poor sanitation and limited access to clean water.^2^ Vaccination is one of the important public health strategies to combat cholera.^3^ The World Health Organization (WHO) has pre-qualified three oral cholera vaccines (OCvs.) to date, including Dukoral® (Valneva), Shanchol™ (Shantha Biotechnics Pvt. Ltd., India), and Euvichol®/Euvichol-Plus**®** (EuBiologics Co., Ltd., South Korea). Shanchol™ was the first of these products to undergo extensive testing and studies to evaluate its stability under different temperature conditions,^3^ which led to specific pre-qualification for use out of the cold chain under Controlled Temperature Chain (CTC) conditions in 2018. CTC is defined as a short-term set of conditions during which certain qualifying vaccines may be stored outside of a traditional +2 to +8°C cold chain for a limited period in a highly monitored and controlled manner. CTC usually permits an exposure of the vaccine to ambient temperatures up to + 40°C for a specific number of days before it is administered to the recipient.^4^

These studies required by the WHO to prequalify a vaccine for use in a CTC typically involve exposing the vaccine to various temperature ranges, monitoring its stability, and assessing any potential degradation or loss of efficacy over time. WHO recommends CTC approaches to ease last-mile vaccine delivery constraints in low and middle income countries (LMICs), but only a few vaccines (the meningitis A vaccine, pneumococcal conjugate vaccine, and human papillomavirus vaccine) are currently WHO-prequalified for CTC use, and despite potential system benefits, wide adoption remains limited due to limited experience and the need for stronger evidence.^5^ In 2023, after reviewing stability and safety study results, WHO’s Strategic Advisory Group of Experts on Immunization (SAGE) acknowledged the public health benefits of applying a CTC strategy to EuBiologics’s Euvichol**®** and Euvichol-Plus**®** OCVs, recommending its use in an interim off-label capacity.^6^

The more standard +2° to +8°C cold chain requirements for vaccine storage and transportation require the availability of refrigerators, freezers, and constant management of ice packs for vaccine transport. This can pose challenges for the campaign team, especially in resource-constrained settings.^5,7^ It is therefore important to explore the feasibility and optimization of deploying OCVs following a CTC strategy in a cholera-prone area, like Bangladesh. The major aim of this study was to compare the logistical feasibility and acceptability of deploying OCVs within a controlled temperature chain in contrast to the currently standard cold chain (SCC) practices in settings at high risk of cholera outbreaks. The results will enlighten future vaccination campaigns and contribute to improving cholera prevention measures in resource-limited settings.

## Methods

### Study design

We conducted a qualitative study to understand multifaceted insights, experiences, and perceptions of stakeholders across different levels of the health system in Bangladesh engaged in vaccine delivery, planning, and oversight. This exploratory qualitative study aimed to capture in-depth perspectives rather than achieve statistical representativeness. It explored the practical experiences, perceived operational advantages, and implementation challenges associated with two vaccine delivery strategies: Standard Cold Chain (SCC) and Controlled Temperature Chain (CTC), used for administering the oral cholera vaccine Euvichol-Plus**®** (EuBiologics Co., Ltd., South Korea) during the OCV campaign in March 2024 in Southern Dhaka, Bangladesh.

### Study settings

This qualitative study was conducted in the Dhaka South City Corporation, concentrating on Dania, a peri-urban area. This area was selected as it represents a mixed urban-peri-urban context, reflecting the typical complex health system delivery dynamics in Bangladesh. Additionally, this area was selected as a pilot site for CTC vaccine delivery approach during the March 2024 campaign, providing a compatible context to examine implementation experiences in a real-world setting where both SCC and CTC strategies were used. As the study focused on a pilot implementation site, the findings reflect context-specific experiences of vaccine delivery during this campaign.

### Sampling strategy, sample size, and participants

A combined approach of purposive and quota sampling was used in this qualitative research to ensure representation across both vaccine delivery strategies and different levels of the health system in Bangladesh. This approach is designed to capture the implementation challenges experienced by both frontline health workers and policymakers involved in the OCV campaign held in March 2024 in southern Dhaka. All the vaccine recipients received a single dose of the OCV (Euvichol-Plus**®**). Guided by the principle of thematic saturation, data collection continued until no substantially new themes emerged from the interviews. The study included ten participants. Six in-depth interviews (IDIs) were conducted with frontline volunteers (vaccinators, supervisors, and record-keepers) assigned to the SCC (Group A) and the CTC (Group B). Among these, three were assigned to the team in SCC implementation, and three were involved in teams implementing CTC strategy. Additionally, four key informant interviews (KIIs) were carried out with national-level policymakers and implementing partners responsible for planning, decision-making, and oversight of the OCV campaign. Table 1 summarizes the roles and affiliations of the study participants.

**Table 1.** In-depth interview (IDI) participants.

Participant type	Group	Role in OCV campaign	Number of participants
Volunteer	Group A	Vaccinator	1
Volunteer	Group A	Supervisor	1
Volunteer	Group A	Record keeper	1
Volunteer	Group B	Vaccinator	1
Volunteer	Group B	Supervisor	1
Volunteer	Group B	Record keeper	1

*OCV campaign volunteers were from icddr,b, and local health facilities.

### Data collection, tools, process, and quality assurance

A trained qualitative team from International Center for Diarrheal Disease Research Bangladesh (icddr,b) with a public health background, conducted IDIs with volunteers and KIIs with the policymakers, using two semi-structured interview guides (Appendices 1A and 1B). The interview guides were developed based on the study objectives and included questions related to implementation experiences, perceived operational advantages, logistical challenges, and feasibility of the two vaccine delivery strategies. Prior to the data collection, the qualitative research team participated in a full-day training program to ensure consistency and preparedness in qualitative interviewing techniques, understanding of study objectives, and familiarity with the guides. The semi-structured interview guidelines were first developed in Bangla and subsequently translated into English by expert bilingual members of the research team and reviewed by the investigators. The interview guides were then validated through internal expert review and pilot tested in the field with three respondents (one vaccinator, one record keeper, and one supervisor) to assess clarity, flow, and feasibility. The Bangla version of the guidelines was used during field data collection. Based on pilot feedback, minor revisions were made to improve wording, sequencing, and probing techniques. The revised Bangla guideline was then back-translated into English to ensure linguistic accuracy and retention of the original context and meaning before finalizing the tools for data collection, including adaptations tailored to the two vaccine delivery strategies. During the training session, the Group A participants were trained on standard cold chain maintenance. On the other hand, Group B received additional training on the controlled temperature chain. All participants received hands-on training and pilot testing to reinforce the team, build confidence, and ensure protocol adherence. IDIs were conducted to explore volunteers’ perceptions and their day-to-day campaign experiences with both CTC and SCC approaches. On the other hand, KIIs gathered expert opinions on the comparative implementation process of both challenges and ease, operational appropriateness, and logistical challenges they faced while conducting both vaccine delivery strategies. Key informants were selected from the Communicable Disease Control (CDC) unit of the Directorate General of Health Services (DGHS), the Institute of Epidemiology, Disease Control and Research (IEDCR), the Expanded Programme on Immunization (EPI), and the Dhaka City Corporation officials. With participants’ informed consent, all the interviews were conducted face-to-face at the workplace of the individual study participants. Prompts and probing were included as part of the interview guide. Before commencement of the interviews, the research team built rapport with the study participants and provided a brief concept of the study and the reason for doing the research. The duration of each interview was approximately 60 min. All interviews were conducted by a trained qualitative researcher, experienced in public health research. Only the researcher, the research assistant, and the participant were present during the interviews. The research assistant took field notes during the interview sessions to capture contextual observations and non-verbal cues. No participants refused or withdrew from the study, and no repeat interviews were conducted. All interviews were conducted in Bangla, audio-recorded and subsequently transcribed verbatim. Research assistants cross-checked the transcripts for accuracy, and the verified transcripts were translated into English by experienced translators and reviewed alongside the Bangla version to ensure consistency and preservation of context. The qualitative data were analyzed manually using thematic analysis, enabling a holistic assessment of the cold chain strategies. This overall approach facilitated the identification of key patterns and insights and practical functionality of SCC and CTC strategies.

### Data management and analysis

To ensure data confidentiality, all interview transcripts were anonymized by employing unique participant codes and removing identifying information. Only authorized research team members could access the documents. Transcripts were not returned to the study participants. Team reviewed each KII and IDI transcripts carefully, systematic memos were prepared on the data through iterative readings. Data were analyzed using deductive thematic analysis guided by the study objectives.

To develop a codebook, the Principal Investigator and Co-Investigator independently coded the subsets of the transcripts to evaluate coding consistency and identify preliminary themes. Next, inconsistencies were refined by thorough repeated team discussions, resulting in a refined codebook and a thematic framework aligned with the study objectives. The finalized coding framework was applied to all transcripts. Preliminary findings, illustrative quotes and analytic memos were reviewed through the team discussion. Illustrative quotes were first selected collaboratively to highlight key findings and were cross-checked to ensure that the interpretations accurately reflected the participants’ statements, consistency, and contextual meaning. During analysis, themes were also compared across participants involved in SCC and CTC delivery to identify similarities and differences in implementation experiences between the two strategies.

### Reporting standards

The study followed the COREQ (COnsolidated criteria for REporting Qualitative research) Checklist to ensure transparency and rigor and adherence to qualitative research reporting standards.

### Vaccine delivery strategies examined

Initially, vaccines were stored at 2–8°C in the icddr,b cold room. A log book was maintained to track the number and status of vaccine vials in terms of the Vaccine Vial Monitor (VVM) stage and expiry date. Field staff were trained to interpret VVMs and to ensure that only vials with an acceptable VVM status, as per manufacturer guidelines, were used for administration. The vaccines were deployed through two different groups: a traditional approach – standard cold chain (SCC) and another approach adopting a controlled temperature chain (CTC) strategy. The CTC approach allowed the vaccines to be removed from the cold chain and stored at monitored ambient temperatures, ensuring that exposure did not exceed 10 d or 40°C in accordance with recommended guidelines. Vaccine stability was monitored using both Vaccine Vial Monitor (VVM) and Peak Threshold Temperature (PTTI). VVM indicates cumulative heat exposure over time whereas PTTIs detect exposure over 40°C. Both the indicators were used to ensure the vaccine quality. VVM must be checked before administration of the vaccines in both SCC and CTC. In case of SCC changes the color of the Vaccine Vial Monitor (VVM) and also CTC full black color changes, we ensure both the acceptable VVM and CTC status. However, both the vaccines were undergone several studies to evaluate their efficacy and also deployed in multiple countries during reactive and preventive OCV campaigns.^8,9^

In both strategies, insulated vaccine carriers were used to store and protect the vaccines. In Group A, conditioned ice packs were added to the carriers to maintain the standard temperature (+2 to +8°C) up to the point of use. At the end of each day, unused vaccines from Group A were returned to the icddr,b, cold room following SCC practices. The SCC (Group A) completed vaccination over 2 d, with vaccines remaining at +2 to +8°C until use. For the CTC (Group B), vaccines were intentionally removed from cold storage several days before administration to allow monitored ambient-temperature exposure as required for CTC implementation. The overall CTC timeline (≤10 d) therefore reflected the combined staging, monitoring, and vaccination period rather than extended vaccination days alone.

## Results

The study findings were structured around key themes and sub-themes that described operational experiences associated with both SCC and CTC strategies. A comparative analysis to assess and evaluate the relative operational feasibility, advantages, and challenges of SCC and CTC in the OCV delivery.

### Characteristics of study participants

The volunteers held a wide range of educational levels, from higher school to post-graduate degrees. Their vaccine-related working experience varied from 2 to 12 y, including responsibilities primarily in hands-on fieldwork, supervision, and community engagement in OCV campaigns. However, key informants had more exposure, with a range of 5 to 19 y of involvement in the vaccine-related programs. All the key informants occupied senior leadership roles within their respective institutions, and their roles encompassed policy designing, microplanning, monitoring, and supervision of OCV campaigns conducted across various geographic settings in Bangladesh.

All participants had prior exposure to OCV campaigns conducted within the SCC chain system. However, none of them had experience with the CTC strategy, which was an essential and central aspect for assessing their readiness and perceptions regarding potential adoption of CTC implementation in the future. Tables 1 and 2 show the profile of all the study participants.

**Table 2.** Key informant interview (KII) participants.

Participant type	Role	Affiliation	Number of participants
Key Informant	Immunization Programme lead	CDC, DGHS	1
Key Informant	Surveillance Expert	IEDCR	1
Key Informant	EPI Manager	Expanded Programme on Immunization	1
Key Informant	Urban Health Coordinator	Dhaka South City Corporation	1

### Implementation challenges of the standard cold chain strategy

#### Inefficiency in maintaining the cold chain

While Bangladesh has adopted freeze-free vaccine carriers to counter risks of freeze damage from vials coming into contact with ice packs, the immunization system continues to be challenged logistically by the conventional cold chain. An important issue in hilly or remote areas is the transportation of heavy loads (up to 10 kg each). Porters often must carry multiple vaccine carriers at the same time, significantly increasing their physical labor and complicating vaccine delivery. According to a national-level key informant,

A freeze-free vaccine carrier is larger and heavier as it carries four icepacks, and with the vaccines inside, each carrier weighs about 8 to 10 kg, which a porter often has to carry, adding up to around 80 kg in total … This is a significant challenge for us. - (P01_ KII _ NAL_27062024, 5 y of experience)

#### Inadequate operational and logistical capacity for hard-to-reach areas

Most study participants reported that lengthy travel times combined with frequent power interruptions made it difficult to condition and maintain ice packs properly and sustain the standard cold chain. This becomes a significant challenge in delivering vaccines to hard-to-reach areas. A national-level KII informant highlighted noting on this issue,

We face major challenges when we need to deliver to reach hard-to-reach areas. Sometimes it can take two to three hours to reach some areas, and due to frequent electricity interruptions, ice conditioning does not work properly. - (P03_ KII_ NAL_14062024, 3 y of experience)

#### Difficulties in temperature management

Study participants reported operational challenges in maintaining the prescribed SCC range of +2° to +8°C, particularly when they transfer the vaccines from primary storage to vaccine carriers. While VVMs were used to assess cumulative heat exposure and determine vaccine usability, they did not detect exposure to freezing temperatures. This issue continues to pose a notable concern, especially when carriers are over-packed with ice or when the chance of ice packs coming into direct contact with vaccine vials. Several participants also raised concerns that, despite using freeze-preventive carriers, the possibility of accidental freezing still existed. Although these potential concerns were raised, no dedicated freeze indicators (e.g., freeze tags) were reported to have been used during the campaign. One of the volunteers from Group A described,

The vaccines remain effective only if kept within the proper temperature range. If the temperature drops too low for a long time, the vaccines can be damaged. Thats why we need to monitor the temperature frequently. - (IDI _Vol_ S_ GA_ 06062024, 12 y experienced)

### Implementation challenges of the controlled temperature chain strategy

#### Lack of experience with the CTC strategy may impede proper adoption

Most national-level key informants predicted that limited familiarity with and practical knowledge of CTC administration among the decision-makers would hinder the adoption of this strategy. Participants also suggested targeted awareness-raising, structured knowledge sharing, and context-specific operational evidence to support informed decision-making. Pointing out this issue, one of the participants highlighted the necessity for a detailed protocol outlining the procedures to guide implementation.

Since CTC is a new strategy to us, it should be maintained through a proper protocol, outlining the proper way and individual roles and responsibilities, such as who will do miking [announcement] and who will feed [administer] the vaccine. Thats essential for successful implementation and helps address our knowledge gap. -(P03_KII_NAL_14062024, 3 y experienced)

#### Perceived risk of exceeding the allowed temperature threshold

Concerns were raised about the required temperature remaining below 40°C during transportation and deployment, particularly in high-temperature regions like North Bengal during the hot season (can often rise to 36–42.7°C).

One of our Volunteers who worked in the CTC group perceived,

In the CTC strategy, vaccines remain usable for up to 14 d if temperatures stay below 40°C. Since no place in Bangladesh requires 14 d to reach, transport isn’t a problem. The real concern is whether weather conditions will consistently stay under 40°C. - (IDI_ Vol _ RK1_ GB_ 02062024, 12 y experienced)

Although the vaccine used in this campaign was allowed only 10 d in CTC, this respondent may have been familiar with another OCV vaccine, which has a CTC period of up to 14 d.

### Controlled temperature chain (CTC) vs. standard cold chain (SCC): comparative insights into vaccine delivery strategies

#### Reaching dose targets: parallel performance and efficiency of CTC and SCC strategies

Both Group A and Group B completed the deployment of 500 vaccine doses within 2 d successfully, which is consistent with the administrative data collected during the campaign and listed in Table 3, including the number of individuals vaccinated per day and the time invested per group.

On the first day, it took longer, and fewer vaccines were deployed; on the next day, it was quicker and more vaccines were administered. - (IDI_ Vol _S _Group A_06062024, 12 y experienced)

**Table 3.** Day-wise performance and time invested for each vaccination session.

	Number of vaccines deployed on Day 1	Time invested in Day 1	Number of Vaccines deployed on Day 2	Time invested in Day 2	Total number of vaccines deployed	Total time invested for vaccine deployment
**Group A (SCC)**	225	8 h47 min	275	9 h17 min	500	SCC = 18 h 4 min
**Group B(CTC)**	225	9 h56 min	275	6 h9 min	500	CTC = 16 h 5 min

(*We have calculated the time from the time of vaccination of the 1^st^ recipient to the time of vaccination of the last recipient of a day).

#### Easing transportation and logistics management

Bangladesh has been utilizing freeze-preventive vaccine carriers as part of the standard cold chain system. The traditional cold chain requires additional logistic support, icepack conditioning, and carrying them. As a result, compared to the SCC, the CTC approach offers a more practical and operationally convenient alternative feature for vaccine delivery. Study participants, from their experience in operating in hard-to-reach areas, described the CTC strategy as more practical and convenient than the SCC. CTC strategy removed the icepack requirement, which enabled them to carry a larger number of vaccine doses, decreasing distribution time and extending coverage capability during the campaign. One of the vaccinators noted,

Unlike the cold chain, CTC is easier to carry … We don’t need to use icepacks in the vaccine carriers for this approach. Its more convenient for campaigns outside Dhaka, as CTC allows for carrying more vaccine doses and requires fewer staff to handle compared to the cold chain. - (IDI_ Vol_ RK 2_ GB_30052024, 2 y experienced)

This reflects how the CTC strategy can reduce logistical complexities and resource demands, enhancing support in improving vaccine delivery to hard-to-reach areas. Echoing the vaccinators, one of the national-level key informants noted,

(In CTC Strategy) if the temperature can be maintained within 40 degrees, it will reduce the workload, increase chances to reach a larger number of vaccine recipients, and allow more vaccines to be transported; ultimately reducing the required time compared to the [standard] cold chain strategy. - (P01_KII_ NAL_ 27062024, 5 y experienced)

#### CTC allows extended storage duration beyond SCC limits

OCVs can be stored for 10 or 14 d, depending on the product, at temperatures below 40°C, as part of approved CTC conditions, which can be advantageous for prolonged campaigns and outreach in underserved regions. One of the volunteers shared his thoughts on the vaccine damage due to the interruption of the cold chain and suggested a way to prevent this.

When we did the vaccination campaign during 2014 and 2016, an entire box of vaccine got damaged, which resulted in the end of the vaccination. But if the cold chain had not needed to be maintained, then this problem would not have occurred. - (IDI_ Vol_ S_ GA_ 06062024, 12 y experienced)

#### Lower human resources requirement for the CTC strategy compared to the SCC strategy

Almost all the study participants consistently emphasized that the CTC strategy reduced human resource involvement compared to the SCC strategy. Volunteers managing SCC reported that frequent temperature monitoring and ice pack handling for this strategy, the workload has increased. On the other hand, CTC was observed as less labor-intensive, providing better operational ease during campaign delivery.

CTC activities seem much easier to us. Earlier, for the cold chain, maintaining temperature, conditioning icepacks, frequent monitoring wasted time, and required more staff. But for CTC, since we don’t need these, a lot of hassle has automatically gone. - (IDI_Vol_S_GA_06062024, 12 y of experience)

#### Cost reduction and efficiency

The study participants reported that CTC strategy implementation significantly reduced costs by minimizing the requirements of human resources and electricity, as it reduced dependency on both extensive monitoring and traditional refrigeration before the final days of vaccine administration. Additionally, they perceived overall CTC strategy is less complex as it lowered transportation costs and simplified logistics compared to the traditional cold chain strategies. CTC strategy facilitated vaccine management efficiently within a 10 d period, which is considered an ideal timeframe for vaccine deployment in the context of Bangladesh. Besides, several participants perceived that, compared to the traditional cold chain strategy, the CTC approach could enhance the efficiency of vaccine deployment and ease of storage, particularly in resource-limited areas.

These views were based on their first-hand experience during the March 2024 campaign, though some also reflected expectations for future use based on comparisons with prior campaigns using the SCC approach. In this context, the majority of the key informants from different stakeholder organizations provided feedback similar to this statement,

If we administer CTC, a huge amount of money can be saved since it does not involve electricity use store the vaccine… . Less human resources, transport, and logistic support were required, which saves a lot. - (P03_KII_NAL_14 JULY 2024, 3 y of experience).

#### Reducing vaccine wastage risk

Almost all the study participants highlighted that the strict temperature management of the SCC strategy (+2 to +8°C) required frequent monitoring to prevent freezing or heat damage, increasing workload and perceived risk of vaccine wastage. In contrast, the CTC strategy was perceived as reducing the likelihood of cold chain breaches and simplifying field implementations. One volunteer shared this comparative experience, emphasizing the operational advantages of CTC.

For administering SCC, we had to check temperatures constantly between 2–8°C to avoid freezing or wastage. On the other hand, for CTC, we faced no such issues, and vaccine wastage was zero. - (IDI_Vol_RK 3_GA_ 09062024, 7 y of experience)

Echoing frontline experiences, national-level key informants also emphasized that the wider temperature range offered by the CTC strategy (up to 40°C) may reduce the risk of vaccine wastage. They noted that vaccine wastage would be unlikely under appropriate implementation, but would occur if the temperature limits were exceeded or guidelines were not followed. This was perceived as an operational advantage of CTC compared to SCC, particularly in settings where cold chain maintenance is challenging.

Since the temperature has to be maintained within 40°C for the CTC strategy, I do not see much risk of vaccine wastage unless the temperature increases more than 40°C, such as direct contact with sunlight. - (P01_KII_ NAL_ 27062024, 5 y of experience)

Table 4 presents an overall comparative insight between SCC vs. CTC below:

**Table 4.** Distribution of different indicators between the SCC strategy and the CTC strategy.

Indicators	SCC Strategy	CTC Strategy
Human Resources	Requires more manpower to prepare ice packs, carry heavy vaccine carriers, and monitor temperature	Fewer manpower required as vaccines can be stored at ambient temperatures up to 40°C and carried without ice.
Workload and time spent	Intensive ice pack preparation and management increase workload and may delay the vaccine deployment	Eliminates the need for a conditioned ice pack supply, which reduces time and the workload of carrying heavy vaccine carriers, enabling faster and efficient vaccine delivery
Required Skill Level	Requires comprehensive training for cold chain management and temperature monitoring	Need the ability to monitor the PTTIs (Peak Temperature Threshold Indicator), and track the duration of the CTC excursion.
Poor resource settings	Depends on uninterrupted electricity and logistical support, which may be absent or insufficient in poor resource settings Requires 2–8°C storage with proper conditioning of ice packs and continuous monitoring	Reduces reliance on electricity for refrigerators and ice pack freezers during the final 10 d, allowing the storage at ambient temperatures Offers the opportunity to avoid the cost and labor of expensive cold storage during the final days and “*last mile*” of delivery
Coverage in the hard-to-reach area	Cold chain constraints, including transport and storage, can limit the vaccine coverage in remote areas due to complex management and time limits for sustaining +2 to +8°C conditions.	Allows carrying vaccines in lighter containers using local and public transportation to coverage in remote areas, and provides more time flexibility
System and operational challenges	Temperature breaches may happen despite proper preparation, risking vaccine discard. High wastage risk due to strict adherence to temperature control and possible freeze exposure.	Ambient temperature in Bangladesh rarely exceeds CTC limits (40°C), enhancing its operational reliability. Low wastage risk since strict temperature management is not needed.
Potential implementation and interpretation challenges	More familiar within the existing EPI system, as the conventional vaccine delivery approach, with established cold chain procedures.	Risk of misinterpreting CTC instructions, including confusion between VVMs and PTTIs. Inadequate training or weak monitoring may increase field-level operational errors and potentially compromise vaccine potency.

## Discussion

The research focused on the expected and actual problems and advantages of using the CTC strategy for giving oral cholera vaccines in a high-risk area in Bangladesh. The information came directly from key people who were interviewed soon after they had completed the vaccine campaign using both the CTC and SCC approaches. The results underscored significant logistical challenges remaining with the traditional cold chain strategy, and the promising benefits of the CTC strategy in overcoming these barriers.

Maintaining the strict +2 to +8°C temperature range and transportation required by the SCC strategy during vaccine deployment was challenging due to factors such as large vaccine carriers made heavy with ice, frequent electricity outages disrupting cold chain equipment. Volunteers and key informants attributed vaccine distribution inefficiencies to logistical challenges: the frequent conditioning and replacement of icepacks, continuous temperature monitoring, and the need for extra personnel to handle the increased workload, which were similar to earlier studies.^5,10^ These elements ultimately increased both the time and expense of vaccine activities and risked limiting coverage in hard-to-reach areas, which restricts the scope and efficacy of immunization campaigns. Participants specifically linked these operational challenges to their recent experience during the March 2024 OCV campaign. They reflected on similar issues, including the time-consuming process of conditioning icepacks, the need for icepack replacement after a time of interval, and the physical strain associated with transporting insulated vaccine carriers, linked with their previous experience with the cold chain strategy. Study participants gave feedback based on their previous experiences and offered suggestions for future planning, particularly to overcome the challenges of reaching difficult locations.

However, the CTC strategy was perceived as enabling these limitations and potentially facilitating improved vaccine reach. The study found that CTC alleviates logistical challenges in areas with inadequate infrastructure or inconsistent electricity by reducing dependency on refrigeration, ice packs, and continuous temperature monitoring, as it allowed vaccines to be stored at ambient temperatures up to 40°C for 10–14 d, depending on the vaccine. This adaptability has the potential to enhance vaccine coverage, particularly in remote regions where the cold chain is challenging to preserve.^11^ Additionally, the CTC strategy provided cost-saving opportunities by reducing the requirements for costly cold storage and ice production infrastructure, and reducing vaccine wastage risk due to temperature fluctuations,^11,12^ as emphasized by both frontline workers and national-level stakeholders. These perspectives helped to contextualize the feasibility of CTC and SCC strategies for OCV programs, particularly in low‑ and middle-income countries.

However, the adoption of the CTC strategy was not without challenges. Key informants reported that limited experience with the CTC strategy, insufficient knowledge to operational protocols, and inadequate awareness among policymakers might slow its widespread adoption.^11^ Some participants also expressed concerns that improper implementation of CTC protocols, such as exceeding allowable exposure duration, exposure to direct sunlight, or confusion between VVMs and PTTIs, particularly in settings with inadequate training or weak monitoring systems, could potentially compromise vaccine potency and increase the risk of vaccine wastage. In contrast, SCC was described by some participants as the established and routinely used system within the EPI program in Bangladesh, reflecting familiarity with existing programmatic practices and standard cold chain monitoring procedures. However, no strong overall preferences for SCC over CTC was observed. Further research should focus on supporting the optimization and broader adoption of the CTC strategy, rather than re-establishing vaccine stability, which was already well-documented. Strengthening CTC adoption will require clearer operational guidance, context-specific planning, improved training, and reinforced monitoring systems to ensure correct implementation and minimize operation errors. Solving these issues through proper planning may support the broader and more effective use of the strategy.

The study had several limitations. It focused mainly on experiences during a small-scale vaccination drive in a peri-urban area of Dhaka. Because of this limited focus, the findings might not fully reflect the real-world logistical and operational problems faced in very rural or remote areas, or in places with different weather conditions.

The small number of participants and the specific way they were chosen (purposive selection) mean that the results have limited generalizability; they may not apply to wider populations. Therefore, the findings should be treated as exploratory insights rather than as definite, proven evidence. Future research is needed to be more comprehensive, incorporating a stronger mix of qualitative and quantitative data to fully assess the CTC strategy’s effectiveness, operational feasibility, cost-efficiency, and potential for use on a large scale (scalability). Based on the results, they suggested that the CTC strategy has the potential to help vaccinators reach every child, even those in hard-to-reach areas, by easing logistical burdens and reducing the demand for human resources. This approach strongly supports Bangladesh’s broader goal of complete immunization coverage.

In conclusion, this qualitative study suggests that the CTC strategy may offer operational advantages compared with the traditional Standard cold chain strategy. CTC reduced logistical challenges in vaccine delivery, particularly in resource-limited and hard-to-reach settings. These findings highlight the potential operational advantages of CTC in improving vaccine delivery efficiency. However, further research is needed to assess its scalability, operational impact, and economic implications within Bangladesh’s health system.

## Supplementary Material

Supplementary_data.docx

## Data Availability

De-identified analytical data set will be made available upon requests directed to the Research Administration of the icddr,b, Shiblee Sayeed, at Shiblee_s@icddrb.org. Only after approval of a proposal, data can be shared through a secure online platform. Approval of the proposal will be subject to scientific review by the institutional review board at icddr,b. Sharing of data will also be subject to the published data access rules of the icddr,b. The requestor will need to sign a standard data access agreement required by the icddr,b.

## Patient and public involvement

The public were involved in the design, conduct, reporting, or dissemination plans of this research.
