# Supplementary material for: Lessons learned from the deployment of oral cholera vaccine by maintaining the controlled temperature chain (CTC) in an urban setting, Bangladesh
Source: Hum Vaccin Immunother. 2026 Jul 23;22(1):2705004. doi: 10.1080/21645515.2026.2705004 (PMC13398100; doi:10.1080/21645515.2026.2705004)
Supplement: Supplementary_data.docx [file KHVI_A_2705004_SM1801.docx]

**Appendix 1A**

| **Guideline for KII with Policy Makers** |
| --- |
| ***Guidelines for interviewers:***  *The topics and questions below should be used to guide the Key Informant Interviews (KIIs). Keep in mind when conducting the KII to respond to the answers provided by the respondents by asking additional questions or adapting to more appropriate questions. The goal of the KII is to engage in interactive, open dialogues.*  *[The descriptions appearing in these grey boxes are for the research assistant only and will not be read to participants.]* |
| *Before the interview:*   1. *Thank the individual for agreeing to talk with you.* 2. *Read the consent form.* 3. *If the individual agrees, sign two copies of the consent form; give one to the interviewee and retain one for the record.* 4. *Say: “I want to thank you for agreeing to share with me some of your thoughts. I have read the Informed Consent form to you, so you now know what this study is about. Are there any questions before we begin?”* 5. *Explain that there are no right or wrong answers, and that they may decline to answer any questions. They may also stop the interview at any time, if they do not wish to continue. Request permission to begin recording the interview.* 6. *Switch on audio recorder* |

|  | | | |
| --- | --- | --- | --- |
| ***Section #1: Background and warm up***  *In this section, participants/respondents will be asked some ice-breaker questions about themselves and their work in general.* | | | |
| ***Core question*** | | | ***Probe*** |
|  | Let’s begin by learning more about you and your work. | | What is your highest level of education? |
|  | Can you tell me more about your work. | | A. What is your designation?  B. How long have you been working in this position? |
|  | What is your role and responsibilities in relation to OCV campaigns? | |  |
|  | | | |
| ***Section #2: Awareness of vaccine delivery challenges and CTC strategy*** | | | |
| ***Core question*** | | ***Probe*** | |
| 1. | Do you have direct prior experience with delivering oral cholera vaccines through a vaccination campaign? | 1. If yes, can you tell me in what capacity you were involved, and 2. how often? | |
| 2. | What are some delivery challenges that you have directly experienced or witnessed during OCV vaccination campaigns? | *COLD CHAIN*   - *Maintaining the required temperature from 2°C to 8°C* - *Conditioning ice to avoid freezing* - *Refreshing ice supplies mid-day*   *TRANSPORTATION LOGISTICS*   - *Carrying vaccine, ice & supplies* - *Navigating rough roads and trails* - *Managing the vehicle* - *Getting out and back in one day*   *FORECASTING / MICROPLANNING*   - *Reaching an active population* - *Estimating the quantity  of vaccine to bring* - *Access to hard to reach populations* - *Keeping wastage to a minimum*   *OTHER: (please elaborate)*  *(Do not ask any leading question, listen to her/him first, if she/he miss anything from the above list, please raise the CATEGORY only,)* | |
| 3. | What is your understanding of the CTC strategy? | What are the main characteristics of a CTC strategy for OCV?  *DURATION*   - *Removal of vaccine from cold chain for up to a* ***specific number of consecutive days*** - *SAGE currently approved off-label* ***10 days*** *of CTC*   *TEMPERATURE*   - *Use of vaccine in ambient temperatures up to 40°C*   *MONIROTING*   - *Monitoring the specific number of CTC days is required to ensure maximum duration is not exceeded* - *Monitoring of upper threshold temperature with a PTTI (peak threshold temperature indicator)*   *RISKS/BENEFITS*   - *Cannot return vaccine vials to cold chain* - *Risk of increased wastage* - *No need for conditioned ice packs* - *Reduced risk of undetected freeze damage* - *Reduced risk of undetected heat damage*   *OTHER (please elaborate)*  *(Do not ask any leading question, listen to her/him first, if she/he miss anything from the above list, please raise the CATEGORY only)* | |
| 4. | According to you, in what ways and how much the vaccine delivery challenges mentioned earlier can be eased by a CTC strategy for OCV? |  | |

|  | |
| --- | --- |
| ***Section #3: Decision-making criteria/requirements*** | |
|  | ***Core question*** |
| 1. | What ideas do you have for making OCV service more effective and efficient in places with limited resources? |
| 2. | What drivers/requirements/criteria would favour putting in place a CTC plan for OCV delivery? |
| 3. | What drivers/criteria would oppose putting in place a CTC plan for OCV delivery? |
|  | What would you need to make a decision about whether to adopt a CTC strategy or not? |

|  | |
| --- | --- |
| ***Section # 4*: Equity and access to vaccination** | |
| ***Core question*** | |
| 1. | Which communities are the most underserved and why? |
| 2. | Which partners play the most critical role in improving access to vaccination and how? |
| 3. | What are persistent evidence gaps which could facilitate decision-making and planning? |
|  |  |
|  | |
| ***Section # 5:*  Policy Implementation** | |
|  | ***Core question*** |
| 1. | How do existing health policies support or hinder the implementation of a CTC strategy for OCV deployment? |

|  | |
| --- | --- |
| ***Section # 6:* Awareness and Support** | |
|  | ***Core question*** |
| 1. | By what means are you typically informed of challenges... “Do you consider this sufficient” |
| 2. | What support mechanisms, if any, have you experienced to facilitate the adoption of innovative vaccine delivery strategies such as CTC? |

|  | |
| --- | --- |
| ***Section # 7:* Collaboration with Stakeholders** | |
|  | ***Core question*** |
| 1. | Which stakeholders are involved in the decision-making process regarding vaccine delivery strategies and how? |
| 2. | Are there collaborations or partnerships in place which address challenges in resource-limited settings? (if so, please briefly describe.) |

|  | |
| --- | --- |
| ***Section # 8:* Monitoring and Evaluation:** | |
|  | ***Core question*** |
| 1. | Do you believe implementing a CTC strategy is feasible and desirable in your setting? (Why or why not?) |
| 2. | What indicators or metrics are useful to you to measure the success or impact of the CTC strategy? |
| 3. | From a policy perspective, what are the perceived benefits and concerns of implementing a CTC strategy for OCV deployment? |
| 4. | If concerns were mentioned, what might be done to address or mitigate these? |
| 5. | Currently, in addition to OCV, there are HPV, TCV, and Hepatitis B birth dose vaccines which also can be delivered through a CTC. Do you consider any of these as needing such a strategy in Bangladesh? |

| *End of discussion* | |
| --- | --- |
|  | Thank you very much for participating and sharing your experiences with me.  Do you have any questions for me before we end the discussion?  Anything else you’d like to add before we close?  Thank you! |

**Appendix 1 B**

**Guideline for IDI with volunteers/vaccinators**

| **Guideline for IDI with volunteers/vaccinators** |
| --- |
| ***Guidelines for interviewers:***  *The topics and questions below should be used to guide the In -depth Interviews (IDIs). Keep in mind when conducting the IDI to respond to the answers provided by the respondents by asking additional questions or adapting to more appropriate questions. The goal of the IDI is to engage in interactive, open dialogues.*  *[The descriptions appearing in these grey boxes are for the research assistant only and will not be read to participants.]* |
| *Before the interview:*   1. *Thank the individual for agreeing to talk with you.* 2. *Read the consent form.* 3. *If the individual agrees, sign two copies of the consent form; give one to the interviewee and retain one for the record.* 4. *Say: “I want to thank you for agreeing to share with me some of your thoughts. I have read the Informed Consent form to you, so you now know what this study is about. Are there any questions before we begin?”* 5. *Explain that there are no right or wrong answers.* 6. *Request permission to begin recording the interview.* 7. *Switch on audio recorder* |

|  | | |
| --- | --- | --- |
| ***Section #1: Background and warm up***  *In this section, participants/respondents will be asked some ice-breaker questions about themselves and their work in general.* | | |
| ***Core question*** | | ***Probe*** |
| 1. | Let’s begin by learning more about you and your work. | What is your highest level of education? |
| 2. | Can you tell me more about your work. | A. What is your designation?  B. How long have you been working in this position? |
| 3. | What is your role and responsibilities in relation to this vaccination campaign? | [use the list below to confirm the correct interpretation of the given response.)  - Inviting community people  - Registration.  - Administer the oral vaccine.  - Supervising the campaign activity.  - Provide information on how to report AEFI to the local health worker.  - Recording AEFI information using appropriate forms.  - Anything else, please specify …………………… |
| 4 | How many previous OCV campaigns have you supported and what were your responsibilities in those campaigns? |  |
| 5. | Have you been involved in vaccination activities for other vaccines? |  |
| 6. | Please describe the optimal condition of a vaccine carrier (vials, ice-packs etc.) for a typical campaign (ie. Standard cold chain) prior to departure from the point where the carrier was prepared for outreach? |  |
| **[The response to this may have been fulfilled by responses to prior questions, and if so, provide the respondent a summary of what you heard and ask if they have anything to add]** | | |

|  | | |
| --- | --- | --- |
| ***Section #2: Support and Challenges***  In this section, questions focus on understanding the infrastructure that supports the campaign activity and the challenges encountered | | |
| ***Core question*** | | ***Probe*** |
| 1. | What are some challenges that you faced to successfully accomplish the campaign activities from beginning to end?  *(Do not ask any leading question, listen to her/him first, if she/he miss anything from the above list, please raise the CATEGORY only,)* | *For group A: (Standard cold chain to maintain)*  *COLD CHAIN*   - *Maintaining the required temperature from 2°C to 8°C* - *Conditioning ice to avoid freezing* - *Refreshing ice supplies mid-day*   *TRANSPORTATION LOGISTICS*   - *Carrying vaccine, ice & supplies* - *Navigating rough roads and trails* - *Managing the vehicle* - *Getting out and back in one day*   *FORECASTING / MICROPLANNING*   - *Reaching an active population* - *Estimating the quantity  of vaccine to bring* - *Access to hard to reach populations* - *Keeping wastage to a minimum*   *OTHER: (please elaborate)* |
|  |  | *For Group B: (CTC strategy, without a Strict cold chain to maintain)*  *CTC MONITORING*   - *Using the PTTI* - *Keeping the vaccines under 40°C* - *Tracking the status of the CTC duration*   *TRANSPORTATION LOGISTICS*   - *Carrying vaccine & supplies* - *Navigating rough roads and trails* - *Managing the vehicle*   *FORECASTING / MICROPLANNING*   - *Reaching an active population* - *Estimating the quantity of vaccine to bring Access to hard to reach populations* - *Keeping wastage to a minimum*   *OTHER: (please elaborate)* |
| 2. | What actions did you have to take to overcome these challenges/gaps? |  |

|  | | |
| --- | --- | --- |
| ***Section #3: Knowledge and attitudes towards OCV delivery with and without CTC*** | | |
| ***Core question*** | | ***Probe*** |
| 1. | Knowledge:  (**Instruction for Interviewers**: ask a, b and c to both the arms)  a. What training and education did you receive before this OCV campaign?  b. What are the storage and handling requirements of the OCV you used for this campaign?c. To what extent has following these requirements adequately been a cause for concern (on a scale of 0/no concern to 5/extremely concerned.)  (**Instruction for Interviewers**: ask d, e, f and g Only to CTC arm)  d. Did you receive training about the VVMs and PTTIs? Please explain these vaccines two-temperature monitoring tools.  e. If the VVMs or PTTI turns black, what are you expected to do?  f. How many days can an OCV vial be kept out of the cold chain? How did you track this?  g. What are the risks for vaccines in a CTC?  Do you think the CTC protocol manages these risks? | 1. Were there any aspects that could have been better explained? Did you feel you knew clearly what needed to be done and how? 2. How did you learn about this? 3. What could help make this easier and why? |
| 2. | Attitude:   1. Do you think delivering the vaccine with a CTC strategy is feasible and/or desireable for future OCV campaigns? Why or why not?   *[If the responder was part of the SCC arm and does not understand sufficiently what CTC is, briefly define CTC. ]* 2. What challenges do you anticipate in implementing a CTC strategy for OCV delivery? 3. What benefits do you anticipate in implementing a CTC strategy for OCV delivery? 4. Given a choice, would you want to use a CTC approach for OCV delivery in the future? Why or why not? 5. Currently, in addition to OCV, there are HPV, TCV, and Hepatitis B birth dose vaccines which also can be delivered through a CTC. Do you consider any of these as needing such a strategy in Bangladesh? |  |
| 3. | Practice:   1. What would you change about the CTC preparation and/or implementation in the future? 2. Have you encountered any challenges in the past related to cold chain requirements for vaccine storage and transportation? 3. Which vaccines did this concern? |  |

|  | | |
| --- | --- | --- |
| ***Section # 4: Performance and efficiency of vaccine delivery*** | | |
| ***Core question*** | | ***Probe*** |
| 1. | In regards to the average number of individuals vaccinated per day: | a. Could you detail what factors affected the immunization rate daily for your team?  b. Were there any administrative or logistical difficulties that impacted the daily vaccination rate? |
| 2. | Average Number of Hours Spent per Vaccination Session: | a. What were the main contributing variables to the length of each vaccination session?  b. Were there any particular efficiencies or inefficiencies noted? |
| 3. | Reach of Zero-Dose Children: | a. Did you come across any children never previously receiving vaccination?  b. What do you believe enabled access to these children this time? |

|  | | |
| --- | --- | --- |
| *Section # 5:* **Vaccine Wastage and Reasons:** | | |
| ***Core question*** | | ***Probe*** |
| 1. | Though vaccine wastage is a normal part of immunization campaigns, did you encounter any during this campaign? If so, what were the causes? |  |
| 2. | What might have been done to avoid this? |  |

| *End of discussion* | |
| --- | --- |
|  | We have reached the end of the interview. Thank you very much for participating and sharing your experiences with me.  Do you have any questions for me before we end the focus group discussion?  Thank you!  *Note: Turn off the audio recorder.* |
